# Buddhism, Christianity, and psychotherapy: A three-way conversation in the mid-twentieth century

**DOI:** 10.1080/13642537.2017.1421985

**Published:** 2018-01-18

**Authors:** Christopher Harding

**Affiliations:** a School of History, Classics, and Archaeology, University of Edinburgh, Edinburgh, UK

**Keywords:** Kosawa Heisaku, religion-psy dialogue, psy disciplines, Carl Rogers, Paul Tillich, KOSAWA HEISAKU, diálogo entre la psicoterapia y la religión, disciplinas psicológicas, Carl Rogers, Paul Illich, Kosawa Heisaku, dialogo religione-psi, discipline psicologiche, Carl Rogers, Paul Tillich, Kosawa Heisaku, dialogue religion-psy, disciplines psy, Carl Rogers, Paul Tillich, Kosawa Heisaku, διάλογος ανάμεσα στη θρησκεία και στην ψυχολογία, σχολές, Carl Rogers, Paul Tillich

## Abstract

This article explores the scope of ‘religion-psy dialogue’ in the mid-twentieth century, via a case study from Japan: Kosawa Heisaku, a Buddhist psychoanalyst based in Tokyo. By putting this case study in brief comparative perspective, with the conversation that took place in 1965 between Paul Tillich and Carl Rogers, the article discusses both the promise and the pitfalls of the modern and contemporary world of ‘religion-psy dialogue’, alongside the means by which specialists in a variety of fields might investigate and hold it to account.

## Abstract (es)

Este artículo explora el alcance del diálogo psicoterapia – religión a mediados del siglo XX, vía el estudio de un caso clinico en Japón: KOSAWA HEISAKU, un psicoanalista budista basado en Tokio. Colocando este estudio de caso en una perspectiva breve, durante una conversación que tuvo lugar en 1965 entre Paul Tillich and Carl Rogers, el artículo presenta dos aspectos: la promesa y los errores del mundo moderno contemporáneo del ‘‘diálogo psicoanálisis – religión’’, así como también los medios por los cuales los especialistas en diferentes campos podrían investigar y llamar a juicio los resultados.

## Abstract (it)

Questo articolo esplora la portata del "dialogo religione-psi" a metà del XX secolo, attraverso un caso proveniente dal Giappone: Kosawa Heisaku, uno psicoanalista buddista di Tokyo. Comparando questa esperienza con la conversazione che ebbe luogo nel 1965 tra Paul Tillich e Carl Rogers, l'articolo discute sia le opportunità che le insidie presenti nel "dialogo religione-psi" che caratterizza il mondo moderno e contemporaneo, oltre che i mezzi che potrebbero essere utilizzati da una varietà di specialisti in campi diversi per ampliare tale dialogo.

## Abstract (fr)

Cet article explore le cadre du « dialogue religion-psy » du milieu du 20ième siècle à travers une étude de cas japonaise du psychanalyste bouddhiste Kosawa Heisaku de Tokyo. En comparant cette brève étude de cas avec la conversation qui eut lieu en 1965 entre Paul Tillich et Carl Rogers, l’article discute à la fois la promesse mais aussi les écueils du monde moderne et contemporain du « dialogue religion-psy » ainsi que les moyens par lesquels des spécialistes venant de champs divers pourraient l’examiner et le tenir pour responsable.

## Περίληψη

Το παρόν άρθρο εξετάζει το πεδίο δράσης του «διαλόγου ανάμεσα στη θρησκεία και την ψυχολογία» στα μέσα του 20^ου^αιώνα μέσα από μια μελέτη περίπτωσης στην Ιαπωνία: Kosawa Heisaku, ένας βουδιστής ψυχαναλυτής στο Τόκυο. Τοποθετώντας αυτή τη μελέτη περίπτωσης σε μια σύντομη συγκριτική οπτική με τη συζήτηση που έλαβε χώρα το 1965 ανάμεσα στον Paul Tillich και στονCarl Rogers, το άρθρο συζητά τόσο τις προοπτικές που ανοίγει όσο και τις παγίδες του μοντέρνου, σύγχρονου κόσμου του «διαλόγου ανάμεσα στη θρησκεία και στην ψυχολογία», παράλληλα με τα μέσα με τα οποία οι ειδικοί από διάφορα πεδία μπορούν να τον διερευνήσουν και να τον αμφισβητήσουν.

What do I do with my distress? This dilemma and its consequences are deeply familiar to psychotherapy. They are also somewhere near the heart of many forms of religious experience. Who am ‘I’ in the first place? Where in my past, in my relationships, in my habits of behaviour, in my character, should I look for the cause of this distress or for my inability to solve it by myself? Who is this distress turning me into? And is this distress ‘mine’ alone? Or is it also a judgement against my society, or my conditions of work – perhaps the whole culture that surrounds me?

The evolution of what Nikolas Rose calls the psy disciplines – primarily psychiatry, psychology and psychotherapy – across the twentieth century incorporated a steady shift towards these sorts of wide-ranging, multi-causal approaches to distress, notably though not exclusively in post-Freudian psychoanalytic, Jungian, Existential and person-centred movements. A similar shift occurred, though for different reasons, within religious traditions like Buddhism – both in Asia and in the West – and Christianity: a fresh willingness to extend religious notions of suffering into the medical and the everyday, and to interpret ‘healing’ more broadly: to include, for example, recovery from, or the religious contextualization of mental illness, addiction and family breakdown. And so we see now books and talks and celebrity figures from the worlds of Buddhism and Christianity making liberal use of psychological terminology, theorizing and case studies. We find pastoral counselling provided across a range of religious traditions, by religious professionals or – increasingly – people with a dual professionalism spanning religion and psychotherapy or counselling. And we encounter the championing of broad benefits for mindfulness meditation, ranging across the personal and inter-relational, the cardiac and neurological, and finally the spiritual and the ethical.

No-one familiar with the introspective, highly psychological writings of the Christian theologian Augustine of Hippo, or the ‘moral therapy’ precursors to scientific psychiatry could argue that dialogue between the religious and the ‘psy’ is somehow uniquely a modern phenomenon. And yet it is in the modern era that we find traditions, individuals, institutions, theories, and practices labelled as ‘religious’ or ‘psy’ being brought quite self-consciously into contact with counterparts across very real (and generally rather recent) professional and hermeneutic divides.

These points of contact, and the social-historical forces that foster them, matter greatly in the present day because of the broad range of ways in which they are capable of influencing everyday life. Since the 1960s, sociologists and social critics like Philip Rieff and Christopher Lasch, followed more recently by Nikolas Rose and Jan De Vos, have worried about the increasingly pervasive political and cultural power of the psy disciplines. Rose has been particularly concerned about a late twentieth-century trend away from traditional, external modes of surveillance and coercion towards more internalized forms, which feel as though they have been freely chosen by us for our own good:

Through self-inspection, self-problematization, self-monitoring and confession, we evaluate ourselves according to the criteria provided for us by others. Through self-reformation, therapy, and the calculated reshaping of speech and emotion, we adjust ourselves by means of the techniques propounded by experts of the soul. (Rose, 1999, p. 11)

To then add religion or spirituality into the mix both complicates matters and raises the stakes. Here, are two powerful but rather malleable sources of epistemic and moral authority – religious obligation or faith, and psychological health or well-being – coming together, and capable of producing ideas and practices and ways of being in the world that may hold enormous sway over people while all but defying objective critique. Encompassing in conceptual scope, deeply personal and complex in its everyday reality – ideas, images, and propositions blending with symbol, myth, emotion, faith, presumed unconscious content, and meditative states – religion-psy dialogue seems to resist adequate description, critique and evaluation via any single meta-language.

No surprise, then, that beyond various assertions of their close compatibility – selflessness as morally desirable *and* psychologically beneficial; important analogies across intrapersonal, interpersonal and transpersonal forms of relating (Schreurs, 2006) – lurk complications, antagonisms (albeit sometimes creative ones), points of confusion, and risks to the safety of patients, clients and parishioners. One could extend these concerns, some of which will be explored shortly, even to instances of cultic manipulation and the partial abdication of government responsibility in providing mental healthcare – on the basis, for example, that for some people meditation or membership of a supportive (religious) community may well be sufficient to address their distress.

If these are all good arguments for seeking to unpick the terms and outcomes of religion-psy dialogue, the question arises of how we ought to do it. The variety and complexity involved prohibits any overarching account, although work in this area has tackled various dimensions of dialogue: the alleged corruption of religion by therapeutic as well as late capitalist forces (Carrette & King, 2004), the broad possibilities where ‘healing’ is concerned (Watts, 2011), and the interface between psychiatry, religion and spirituality (Cook, 2013). In addition to this, by pursuing specific case studies, and then comparing two or more of them, we may find that useful themes and insights emerge: concerning the way in which pioneering thinkers and practitioners exercise power in their work, the nature of the risks to clients that arise as a result, the ways in which clients respond, and the impact upon wider society when hybrid religion-psy ideas make there way out of the consulting room and into wider society (in the form, for example, of everything from professional religious and psychotherapeutic publications to self-help advice for the general reader).

Elsewhere, I have sought to provide a sample framework for how case studies may be carried out and compared (Harding, 2016). In this present article, my aim is to introduce a case-study from mid twentieth-century Japan, and then to suggest the benefits of comparison by showing briefly how some of the themes that arose in Japan find distinct parallels in the United States around the same time.

This comparative or networked (where there are many more than just two) case study approach has the advantage of being less likely to miss the specific historical, cultural and personal dynamics of each instance of dialogue, reminding us that beyond this rather abstract term – ‘religion-psy dialogue’ – lies no concrete reality, but rather a series of discrete realities, which nevertheless also possess important and informative overlapping features.

## Kosawa Heisaku: Buddhism and Psychotherapy

The smash of the plate reverberates around the kitchen. In the silence that follows the little boy looks up to see his father’s fury building. Then out it comes, torrents of recrimination: Why did he do that to a precious plate? Why the hell can this boy never concentrate?

The boy apologizes, means it, but on and on it goes with his father’s anger, until the boy can’t help but shout back: It was a mistake! And I’ve tried to apologize! What more do you want?!

But the boy’s mother, too, is in the room: People are people, my love, and though what you did was truly wrong, never forget that I know you. And I know you can’t help it with things like this – no matter how hard you try.

A sigh escapes the boy’s mouth, his shoulders drop. He dissolves into tears: Thankyou mother. Thankyou … I really am sorry. I won’t do it again.

A young Kosawa Heisaku offered this ‘parable’, as he called it, to his readers in the mid-1930s as a way of helping them to appreciate the difference between two sorts of human guilt (Kosawa, 1931). The father gives rise in the boy to the first sort: guilt rooted in fear of punishment, fuelled and warped by enormous hostility. The mother inspires the second: a guilt that Kosawa preferred to call true, revolutionary ‘repentance’, using a Buddhist term – *zange* – that was to take on enormous significance in Kosawa’s personal and professional lives.

Kosawa was a psychiatrist, and one of Japan’s first psychoanalysts. He was sure that Sigmund Freud had misunderstood both guilt and religion. A religious state of mind, suggested Freud twenty years before in *Totem and Taboo*, is rooted in an attempt to allay guilt over mankind’s primeval slaying of a hated and feared father figure: a long-ago drama that replays itself in the developing psychic lives of young children. But for Kosawa this was not the only – nor the truest – ‘religious state of mind’. For that, one has to look at what the mother generates in the boy, as he stands there surrounded by bits of broken plate, his father fuming a few feet away. *I know you. And I know you can’t help it with things like this*. To become deeply aware of one’s real failings, and then to feel completely met there, known and held by something much greater. Where these twin processes occur, thought Kosawa – whose insights here somewhat parallel those of object relations theorists, Melanie Klein and Donald Winnicott in particular (Ross, 2010) – one finds a liberated state of mind or heart that really does deserve to be called ‘religious’.

The parable may well have had origins in the Kosawa family home. Heisaku was born the ninth of ten children, in a village in Kanagawa prefecture where his family owned rice fields. His father, Takatarō – ‘hawk-boy’ – ran a small bank and dealt in tobacco on the side (Takeda, 1990). Beyond those fields lay Ōyama mountain – known as Aburi-san, or Rainy Mountain, for the heavy dark clouds that regularly burst overhead. Ōyama was a recurring theme in Heisaku’s dreams and free associations: he recalled being struck afresh by its beauty on a trip home after long months away; once he remembered – or perhaps imagined – sitting in a mountainside teahouse festooned with wisteria, opening a Viennese umbrella as the rain began to fall (Kosawa, *Furoido sensei kenkyū nōto*; *yume bunseki*).

Growing up, father and son had relatively little to do with one another. Nine siblings were formidable competition, and the eldest – a girl – was already twenty years old when Heisaku came along, by which time fatherhood for Takatarō may have lost much of its charm. To make matters worse, under Japan’s *ie* (household) system, the first son mattered by far the most in terms of family name and inheritance. Heisaku’s existence was, at least in this social sense, neither here nor there. His eldest brother’s name – 宗栄: Sōei – meant religious glory or honour. Heisaku – 平作 – meant ‘normal crop’.

Other than Kosawa’s early wish to become Prime Minister, which he attributed to the influence of his father’s politics, there is little evidence that Takatarō attended to or shaped the young boy directly in any meaningful way. Kosawa later recalled Takatarō reserving most of his attention instead for the third son of the family, Saburō, who appears to have suffered with behavioural problems. Kosawa resolved to become a psychiatrist one day partly so that he could better understand people like Saburō – how they came to be the way they were, and what might be done about it.

Takatarō’s influence on Kosawa was largely indirect, but formidable nonetheless. He plainly scared his son, who found him overbearing, unpredictable, stubborn and fundamentally selfish (Kosawa, *yume bunseki*). He recalled his father once complaining to him about the cost of Heisaku’s lengthy hospital treatment for a detached retina while in his mid-20s. Despite this being a serious condition, and doubtless a frightening experience, the only fatherly comments that stayed with Kosawa were that one family member shouldn’t be allowed (financially) to destroy the rest. Even as Takatarō lay dying, there was no let up. Kosawa was by this point a fully qualified medical doctor, pursuing a specialism at one of the country’s most prestigious universities. His father simply asked him how long he intended to keep messing around ‘at school’ (Kosawa, *Satogaeri no ki*). Kosawa, for his part, tried to bring what he could of his medical know-how to prolonging his father’s life. He was unsuccessful, and may even have blamed himself for his father’s early death in 1931.

Two things now happened. First, Kosawa started to identify himself with Prince Ajase, who in a famous Buddhist legend set during the lifetime of the historical Buddha imprisons and then kills his father, King Bimbisara. By the summer of 1931, Kosawa had published a mixed psychoanalytic and religious theory of guilt based on this legend, including in it his parable of the smashed plate. Second, Kosawa left Japan in late 1931 to seek out Sigmund Freud. He wrote to his brother Ichirō – the fourth son, who having inherited the family’s money agreed to help finance Kosawa’s trip – that his aim was to get Freud’s approval for his new ‘Ajase Complex’ idea and then return to lay his completed thesis at their father’s grave (Kosawa, 1932).

In Kosawa’s parable, the child’s mother appears by contrast as a saintly figure: capable of great patience and gentleness but also possessing a spiritual gift of sorts, able to see into the depths of the child, meet him there, and nurture a ‘truly religious state of mind’. Kosawa’s own mother, Kon, was a rather more complex figure, and in any case as was common for a family of the Kosawas’ standing, the young Heisaku was mostly looked after as a child not by his mother but by a local girl serving as his nanny. ‘Ichi’ was around ten years old when she began looking after Heisaku, and as one might expect from someone of that age was not entirely committed to the idea of responsible childcare. Missing her friends, she once tied Heisaku to a tree so that he couldn’t wander off while she went out to play. Much later in life, Kosawa’s thoughts used to return frequently to that moment – a sign, thought one of his students, that his idealization of the maternal possessed deep roots not in satisfaction but in longing and loss (Takeda, 1990).^1^

Kosawa was by no means out of the ordinary here. He grew up in a society many of whose men rhapsodized rather than really knew their mothers (Napier, 1996), and who sought to fix the womanly and the maternal as comforting social, emotional and even spiritual categories. She was, nevertheless, a strong presence in his life, ensuring that ‘home’ offered a reassuring resonance and embrace for Heisaku – powerful enough to surface years later in his free associations. He remembered her joy when he used to return home from boarding at his Higher School in Sendai, in the north of Japan. Mother would make *amazake* – a sweet rice drink – to welcome him back. She would smile at him as he licked his cup – ‘like a baby’, he wrote, ‘just moving onto solid food’. Heisaku remembered too the vivid sensation of being in the bath with her (Kosawa, *yume bunseki*).

Kosawa’s early relationships with his father and mother seem to have shaped his attitudes towards both Buddhism and mental health, and to have inspired his interest in seeing the two placed together in a single, salvific system – albeit with Buddhism and psychoanalysis still retaining separate goals and languages and institutions of their own. Helping to set this emerging conversation in train was a Buddhist monk of the Jōdo Shinshū (Shin) sect by the name of Chikazumi Jōkan. Kosawa met Chikazumi while he was at Higher School in northern Japan, encountering via him a highly devotional, emotional form of Buddhism in which intra-familial relationships were understood as furnishing the individual with transpersonal salvific opportunity. Chikazumi came to this realization while lying in bed one day, critically ill, and hearing his father by his bedside quietly wishing his son’s troubles upon himself. It became a moment of conversion: an encounter with compassion so strong and pure that all at once it broke him out of the small, citadel mentality of a young university intellectual, showing him instead a truer, more vital vision of himself – as weak, vulnerable and loved (Iwata, 2011).

The proximate source of this love and compassion was his father. Their ultimate source, for human beings, was the quasi-monotheistic figure of the cosmic Buddha ‘Amida’ – the Buddha of Infinite Light. The thirteenth-century founder of Shin Buddhism, a man by the name of Shinran, had insisted that the limits of human nature and the boundless compassion of Amida were such that a person needed only to recite a short prayer – *Namu*-*Amida*-*Butsu*: ‘Hail to Amida Buddha’ – in order to be saved. This wasn’t some magical formula. Rather, it was an honest and profoundly generative recognition of weakness, such that the very term ‘weakness’ lost its typical, negative connotations.

Shinran went as far as to say that this prayer could only be truly spoken by Amida Buddha himself, working at the deepest level of a person’s subjectivity. As Kai Wariko, a modern Shin poet, put it:

The voice with which I call Amida Buddha

Is the voice with which Amida Buddha calls to me.

For Chikazumi, and soon for Kosawa, the full force of his home life coming into play alongside a new-found devotionalism, modern Japanese family relationships were the means by which Amida’s mercy broke into mundane, linear time, and into mundane, human lives (Iwata, 2011).

To twenty-first century ears, much of this must already sound like psychotherapy of a sort (Ross, 2010). But for Kosawa, the connection only really came when he encountered the writings of Sigmund Freud while at university. He found in Freud a modern-day Shinran: someone who understood human frailty, and appeared to be on a quest to tease out and treat them, bringing all the tools of modern science to the task.

A few months spent in Vienna, with Freud and his circle, failed to change Kosawa’s mind about Freud and the purpose of psychoanalysis, although he did write home to his brother to say that he was a little disappointed with the general level of psychoanalytic practice in Vienna – and was looking forward to getting home to start his own clinic in Tokyo (Harding, 2014).^2^ This he accomplished in 1933, seeing hundreds of clients from all walks of life over the next few years – students, farmers, civil servants, company employees, even a sushi chef, a politician, and a Buddhist monk. From his client records, and from the testimony of those still alive who were treated by Kosawa, we get a sense of the therapeutic fruits of Kosawa’s inner religion-psy dialogue across his early life.


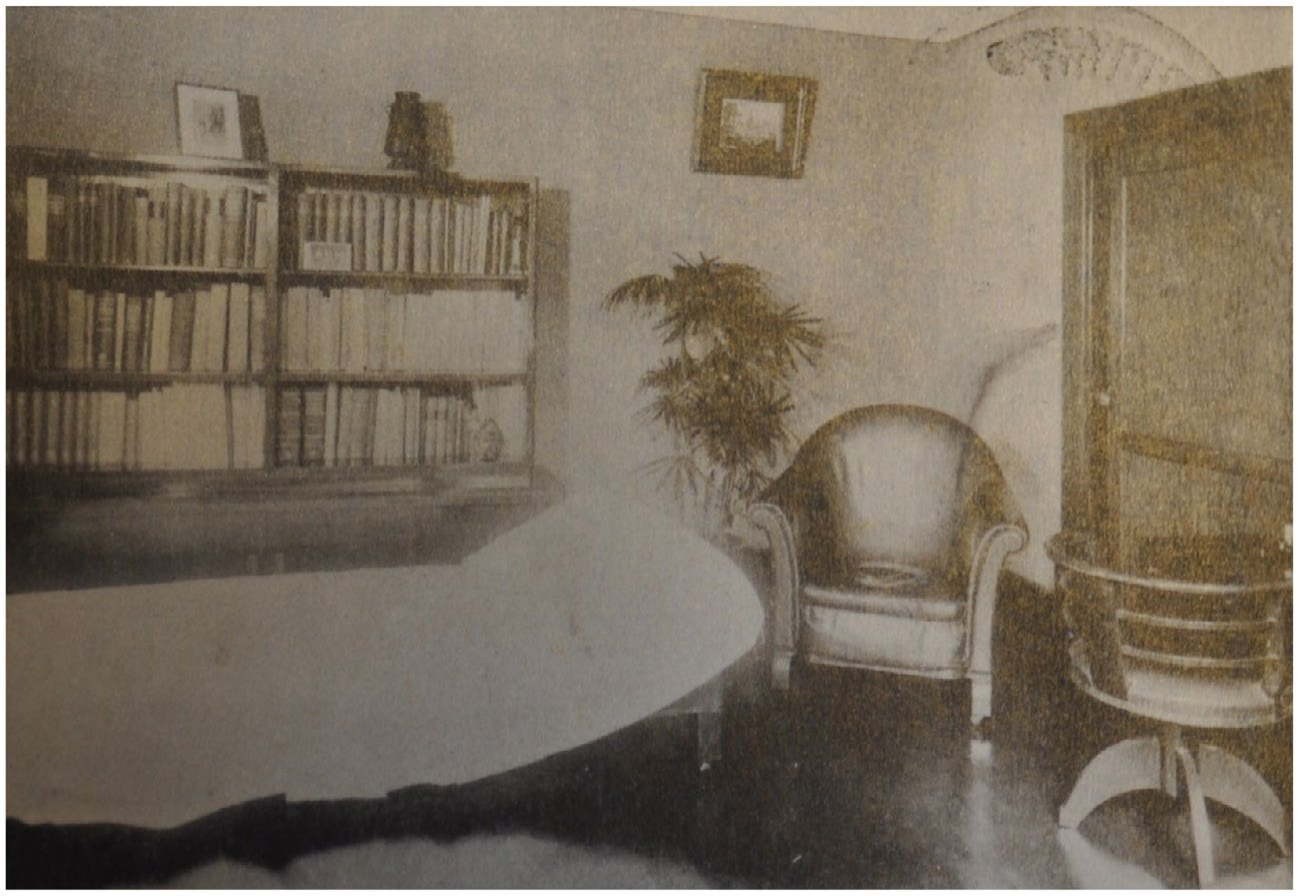
 Kosawa’s impeccably neutral consulting room – Buddhist conversation with interested clients would instead be held next door, once the session had ended.

Perhaps most revealing are the records of what Kosawa called ‘psychoanalysis by mail’. This involved asking clients who couldn’t make it for face-to-face sessions in Tokyo to send him, at regular intervals, two documents. The first was a covering letter, addressed to Kosawa. The second was a written record of a period of solo free association – all that had flashed through their heads when they obeyed the standard psychoanalytic command to allow their thoughts and feelings to go where they will. The first document told Kosawa about the client’s own self-understanding. The second was the really interesting one: it revealed something of what lurked in the client’s unconscious. As therapy went on, Kosawa would hope to see material move from this second document into the first: unconscious elements making their way into conscious awareness.

One client confided in Kosawa that he recalled being embarrassed, as a child, when his parents forced him to wear a girl’s rubber swimming cap at the seaside. Kosawa responded that he hadn’t been embarrassed at all: he had liked it.

Covering up the ears symbolizes castration, which in turn suggests your desire to become a girl as a means of securing affection from your father. What’s more, your recollection and sharing of that memory now may well be a sign of homosexual feelings towards me …

Perhaps the client spilled his morning tea as he read this. Maybe he glanced nervously over the top of the letter at his father sitting across the table. Whatever happened, this was part analysis, part carefully calibrated attempt to nudge a client who was beginning to over-intellectualize the process of therapy into precisely the kind of gentle, helpless humiliation that Kosawa believed Shinran and Freud were agreed was central to the success of religious practice and psychotherapy alike. One day, a client of Kosawa’s reported to him a vivid experience of being momentarily outside of himself, or at least not quite ‘in’ himself in the usual way. Kosawa was overjoyed. *That*, he said, *is the real aim of psychoanalysis. Without it, psychoanalysis as a technique can never survive* (Harding, 2014).

## Religion-Psy Dialogue: Four Emerging Themes

Awareness in the West of the Shin Buddhist sect to which Kosawa belonged has generally been low, despite its considerable size and power in Japan.^3^ Buddhism, and especially Japanese Buddhism, has tended to be associated more with Zen. One of the reasons put forward for this is that in Zen, mid-twentieth-century Westerners found a combination they felt Christianity failed any longer to provide: vivid experience, real inner change, minimal theological baggage. Shin Buddhism’s emphasis on faith and fundamental human inadequacy, by contrast, was too close for comfort to Protestant, particularly Calvinist Christianity. Why travel thousands of miles to a brand new culture – whether literally or in one’s reading – only to find the very thing you were trying to escape?

And yet for all the past and on-going interest in how Zen and psychoanalysis might work together, pioneered in the late 1950s and early 1960s by Erich Fromm and Japan’s famous Buddhist evangelist D.T. Suzuki (Fromm, Suzuki, & De Martino, 1960), Shin Buddhism and psychotherapy – as Kosawa’s experience shows – possess promising commonalities. So it is all the more interesting to see that just five years after Fromm and Suzuki’s work was published, Protestant Christianity and psychotherapy were entering into dialogue via a radio and television studio conversation between the German-American theologian Paul Tillich and Carl Rogers – the latter once having spent time in training for the Christian ministry.


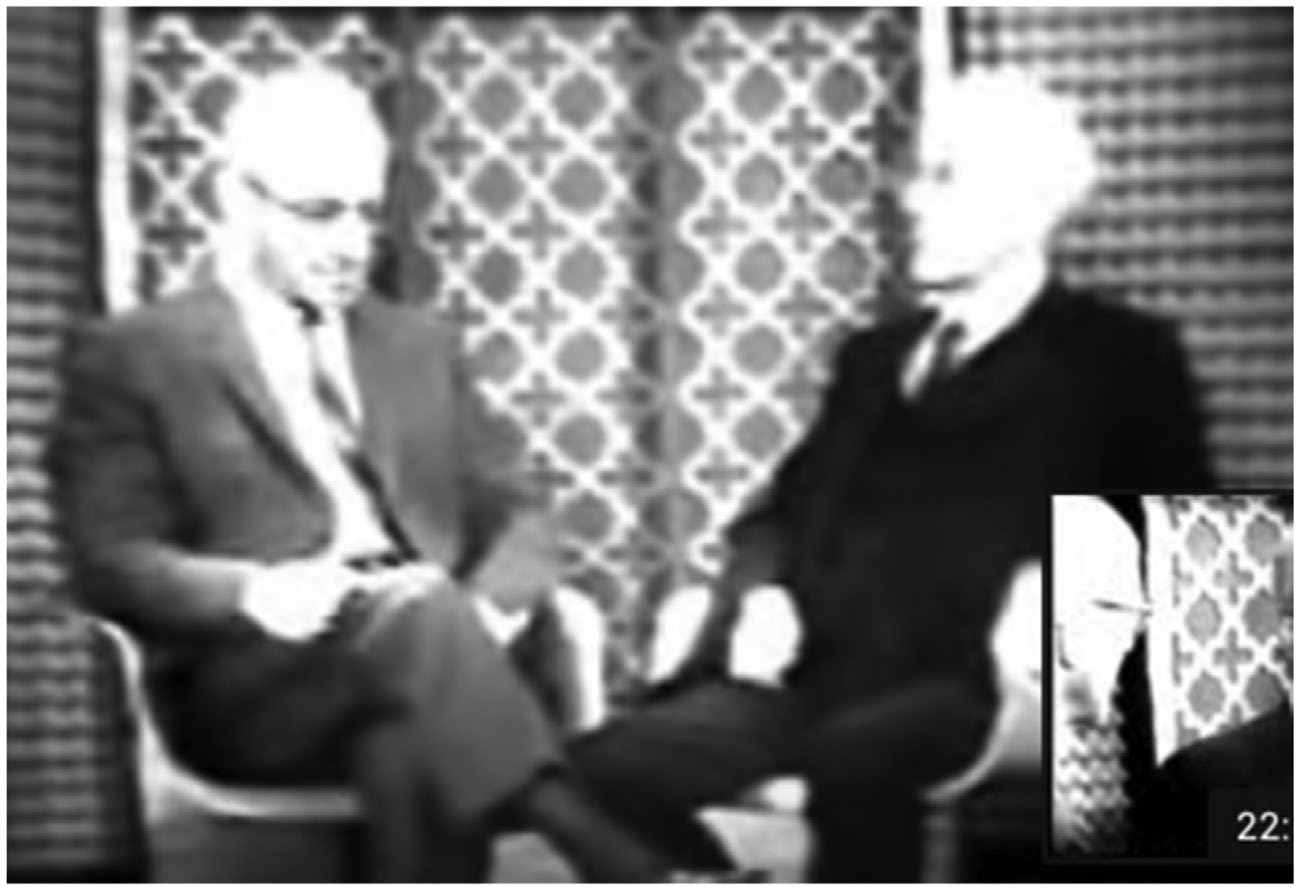
Carl Rogers in conversation with Paul Tillich, filmed for television in 1965.

We may identify four key themes arising in and from Kosawa’s inner religion-psy dialogue and the more literal ‘dialogue’ between Tillich and Rogers: a concern with the damaging social and psychological impact of modernity; a conviction that a profound experience of acceptance lies at the heart of the remedy; a sense that, almost by definition, this cannot be effected by the individual acting alone for his or her own benefit; and a danger – pointed out by critics – that such a remedy, especially where it takes hybrid religion-psy form, risks fostering subjectivities more suited to the totalitarian societies of the recent past in Europe and Japan than their postwar counterparts. It is worth briefly addressing each of these themes in turn.

Kosawa Heisaku was a great critic both of what he saw as pathological individualism in early 1930s Japan and the false community and comfort offered by Marxism and by Japan’s ‘new religions’, the latter striking Kosawa as shallow and manipulative. Kosawa was hardly alone in these concerns: commentators from the novelist Natsume Sōseki through to journalists and philosophers like Watsuji Tetsurō worried about Japan’s social and cultural fabric coming apart under the pressure of successive waves of Western fads and fashions from the late nineteenth century onwards. In the United States, both Paul Tillich and Carl Rogers worried, as Terry Cooper has shown (Cooper, 2006), in similar ways. Tillich wrote about ‘estrangement’: from the ground of our being (a phrase Tillich used frequently for what other theologians called God), from others, and from ourselves. Rogers coined the term ‘incongruence’: the result of steadily concealing parts of ourselves from others as we grow up, in the hope of making ourselves more acceptable, the end-point of which is the partial concealment of ourselves from ourselves (Cooper, 2006, pp. 17–21).

In all three schemes – Kosawa, Tillich, Rogers – we find that acceptance plays a crucial role in countering all this. Not ‘acceptance’ in the everyday sense for which ‘tolerance’ may be the more accurate term, but rather the kind of acceptance that relies on deep knowledge of the person who is being accepted, along with that person’s willingness and ability to, as it were, accept the acceptance. One of the reasons why Kosawa chose a little boy, as opposed to an adult, for his parable may be that a little boy, or girl, might still be at the stage where they have not lost their natural ability to accept acceptance. What Rogers called ‘conditions of worth’, all too clearly communicated in the father’s outburst in the parable, have not yet become completely entrenched.

But here is an important point of complication: where does this acceptance come from? For Rogers, the human realm is the source. For Tillich, the human realm – including family and therapist – mediates an acceptance that comes from somewhere well beyond us. Though there are obvious perils in seeking to compare Kosawa’s with Tillich’s cosmologies, the two share something important in common here. They are convinced that anxiety or other forms of distress stem, in part at least, from our very nature, which in turn is a ‘given’ of existence rather than something we are capable of shaping. Because of this there is always need of what Tillich called grace and what Kosawa understood as the working of Amida’s compassion: some salvific force from without, operating in and through the human.

This places important limits on religion-psy dialogue, in at least two ways. First, though in entirely secular counselling settings one could talk of moments of ‘grace’ (where something powerful and unintended arises), and on that basis much fruitful conversation can happen between the religion and psy spheres, Tillich’s and Kosawa’s cosmologies nevertheless understand the human as something else, or more. So for all their usefulness as bridging concepts between the religious and the psy, ideas like ‘grace’ should not have distinct meanings folded into them as though such things do not matter. Second, in Kosawa’s and Tillich’s cosmologies human limitations extend to our ability to formulate *any* adequate concepts – suggesting an even more fundamental hermeneutic problem for religion-psy dialogue (Hirota, 2011).^4^

Critics of particular instances of religion-psy dialogue have been quick to offer related cautions. We need some way of distinguishing in meditation, argues one, between psychological insights (about us, in the past and present) and spiritual insights (relating to the ‘divine’, for wont of a more appropriately inter-religious term, and our place in it, or relationship with it). We need to avoid religion-psy ‘dialogue’ devolving into the former simply serving the latter – as a provider of high-sounding, inspirational alternative terminologies for what is basically psychology, effectively masking a slide into agnosticism.^5^ Still others wonder how forms of Buddhism and Christianity that resist the idea of ultimate reality having a personal dimension can meaningfully talk about ‘acceptance’. Surely acceptance is a process with a person at either end (Cooper, 2006; Harding, 2016).

Lastly, there is a concern about what kinds of people some forms of religion-psy dialogue helps to create. Kosawa, in his own day, was accused of literally ‘drinking’ his clients: thriving on their tales of distress and even playing the saviour to some extent – unable, despite his theorizing to the contrary, to imagine himself rather than Amida Buddha as the source of a person’s felt acceptance. An American critic, though not especially well informed about Japan, offered the provocative criticism that whereas psychoanalysis in the United States sought to free the individual from the fetters of society, people like Kosawa actively sought to tighten them. If one stands back and seeks to read, at a purely social level, Kosawa’s therapy, then reliant as it was on encouraging an individual to really experience their frailty, inadequacy, and deep need of others it might indeed fit such concerns. Read, however, at a religious or philosophical level, there might seem to have rather more going on: on this reading, *everyone* is heir to the very same constitutional weaknesses, so there can – or at least *should* – be no tyranny of ‘strong’ over ‘weak’. In practice, of course, there was the serious risk of a religious reading glossing, even enabling, the harmful effects described in a social reading. Kosawa was criticized on precisely these grounds by some of his young psychoanalytic trainees.

For Kosawa Heisaku, the fruits of religion-psy dialogue emerged in two simple questions: what is insight, and what does it cost? Good questions, which seem likely to remain with us for some time yet. And there is much to welcome, from this point of view, in the increasing interaction between the religion and psy professions, institutions, ideas and practices – even orientations within individuals. Finding ways to hold such interaction to account, by understanding its sources, claims, motivations and possible implications, is an important task, in which a range of academic and non-academic specialisms have roles to play. This article has sought to sketch out a social-historical and transcultural approach, as just one of many potential angles on this complex modern and contemporary phenomenon.

## Disclosure statement

No potential conflict of interest was reported by the author.

## Funding

This work was supported by the Japan Society for the Promotion of Science; Wellcome Trust and British Academy.

## Notes on contributor

***Christopher Harding*** is a lecturer in Asian History at the University of Edinburgh. He works on the cultural history of modern India and Japan, with a particular interest in religion, spirituality, and mental health.

## Notes

1. Another of Kosawa’s students, Dr Maeda Shigeharu, agrees, suggesting that Kosawa suffered from not receiving as much love from his mother as he needed. Author interview, March 2016.

2. See also Blowers and Yang Hsueh Chi (1997).

3. An important therapeutic exception is Brazier (2007).

4. On some of the complications of this debate, see Hirota (2011).

5. On the difficult relationships across the twentieth century between Christianity and psychotherapy/counselling, see Johnson and Jones (2000).
